# Effects of orally administered *Euglena gracilis* and its reserve polysaccharide, paramylon, on gastric dysplasia in *A4gnt* knockout mice

**DOI:** 10.1038/s41598-021-92013-5

**Published:** 2021-07-01

**Authors:** Masataka Iida, Mark Joseph Desamero, Kosuke Yasuda, Ayaka Nakashima, Kengo Suzuki, James Ken Chambers, Kazuyuki Uchida, Ryohei Ogawa, Satoshi Hachimura, Jun Nakayama, Shigeru Kyuwa, Kozue Miura, Shigeru Kakuta, Kazuhiro Hirayama

**Affiliations:** 1 Laboratory of Veterinary Public Health, Graduate School of Agricultural and Life Sciences, The University of Tokyo, 1-1-1, Yayoi, Bunkyo-ku, Tokyo, 113-8657 Japan; 2 Laboratory of Biomedical Science, Graduate School of Agricultural and Life Sciences, The University of Tokyo, 1-1-1, Yayoi, Bunkyo-ku, Tokyo, 113-8657 Japan; 3 Department of Basic Veterinary Sciences, College of Veterinary Medicine, University of the Philippines Los Baños, 4031 Los Banos, Laguna Philippines; 4 euglena Co., Ltd, Minato-ku, Tokyo, 108-0014 Japan; 5 Laboratory of Veterinary Pathology, Graduate School of Agricultural and Life Sciences, The University of Tokyo, 1-1-1, Yayoi, Bunkyo-ku, Tokyo, 113-8657 Japan; 6 Research Center for Food Safety, Graduate School of Agricultural and Life Sciences, The University of Tokyo, 1-1-1, Yayoi, Bunkyo-ku, Tokyo, 113-8657 Japan; 7 Department of Molecular Pathology, Shinshu University School of Medicine, 3-1-1, Asahi, Matsumoto, Nagano 390-8621 Japan

**Keywords:** Immunology, Gastroenterology, Oncology

## Abstract

*Euglena gracilis* is widely utilized as food or supplement to promote human and animal health, as it contains rich nutrients. In this study, we administered spray-dried powder of *E. gracilis* and paramylon, β-glucan stored in *E. gracilis* cells, to *A4gnt* knockout (KO) mice. *A4gnt* KO mice are a mutant mouse model that spontaneously develops gastric cancer through hyperplasia-dysplasia-adenocarcinoma sequence in the antrum of the stomach, and we observed the effects of *E. gracilis* and paramylon on the early involvements of *A4gnt* KO mice. Male and female 10-week-old *A4gnt* KO mice and their age-matched wildtype C57BL/6J mice were orally administered with 50 mg of *E. gracilis* or paramylon suspended in saline or saline as a control. After 3-week administration, animals were euthanatized and the stomach was examined histopathologically and immunohistochemically. Gene expression patterns of the stomach, which have been reported to be altered with *A4gnt* KO, and IgA concentration in small intestine were also analyzed with real-time PCR and ELISA, respectively. Administration of *Euglena* significantly reduced the number of stimulated CD3-positive T-lymphocytes in pyloric mucosa of *A4gnt* KO mice and tend to reduce polymorphonuclear leukocytes infiltration. *Euglena* administration further downregulated the expression of *Il11* and *Cxcl1* of *A4gnt* KO mice. *Euglena* administration also affected IgA concentration in small intestinal contents of *A4gnt* KO mice. Paramylon administration reduced the number of CD3-positive lymphocytes in pyloric mucosa of *A4gnt* KO mice, and downregulated the expressions of *Il11* and *Ccl2* of *A4gnt* KO mice. Although we found no significant effects on gross and microscopic signs of gastric dysplasia and cell proliferation, the present study suggests that the administration of *Euglena* and paramylon may ameliorate the early involvements of *A4gnt* mice through the effects on inflammatory reactions in the gastric mucosa. The cancer-preventing effects should be studied with long-term experiments until actual gastric cancer formation.

## Introduction

Disease prevention and early disease management by food or food components with health-promoting activities along with their nutritional benefits have recently been attracting attention and the term “functional food” is now widely accepted^1^. Various functions have been reported including the reduced risk of coronary heart disease^2^ and the beneficial effect on irritable bowel syndrome^3^. Anti-tumor effect is also one of the growing focused effects. To date, a broad range of examples has been extensively characterized, including eicosapentaenoic acid (EPA), docosahexaenoic acid (DHA), probiotics, phytochemicals, lycopene and β-glucans^4^.

*Euglena gracilis* (*Euglena*) is a unicellular photosynthesizing green alga living in fresh water. In addition to being enriched in nutrients such as vitamins, minerals, amino acids, and fatty acids at high concentrations, it has no cell wall and is highly digestible. Moreover, it has been previously shown to exemplify a number of health-enhancing activities as supported by few studies^5–7^. Therefore, *Euglena* is now widely utilized as a food or supplement to promote human and animal health. One of the functional ingredients responsible for the health effects of *Euglena* is paramylon, a β-1,3-glucan comprising around 20–70% of *Euglena* on a dry weight basis. This stored polysaccharide has been reported to possess various effects on the host including immunomodulating effect^6,8–13^.

β-glucans from multiple sources i.e. mushroom, bacteria, oats, etc. have been well established to exhibit a pronounced anti-inflammatory and anti-tumor efficacies^14,15^, while the anti-tumor activity of paramylon or *Euglena* is still poorly understood. Previous studies demonstrated that paramylon from *Euglena* prevents preneoplastic lesions in the mice large intestine, and that another species, *Euglena tuba*, suppresses metastasis in human lung and breast carcinoma cells. However, it still remains unknown whether paramylon or *Euglena* have preventive effects on a precursor of gastric cancer^16,17^. Thus, in the present study, we investigated the effects of oral administration of *Euglena* and paramylon on the early involvements of gastric carcinogenesis in a genetic mouse model.

In this study, we employed *A4gnt* knockout (KO) mice as an experimental model. *A4gnt* KO mice are deficient in α1,4-*N*-acetylglucosaminyltransferase (α4GnT), a glycosyltransferase responsible for the biosynthesis of α1,4-*N*-acetylglucosamine capped *O*-glycans (αGlcNAc) in gastric gland mucin^18^. This mutant mouse model spontaneously develops gastric cancer through hyperplasia-dysplasia-adenocarcinoma sequence in the antrum of the stomach^19^. At 10–20 weeks of age, the age used in the present study, *A4gnt* KO mice show precancerous lesion of gastric cancer, dysplasia.

In addition, we also measured the concentration of IgA in tissue and contents of small intestine of the mice, because many β-glucans have been reported to have immunostimulatory effects through host innate immunity^8^.

## Results

### Effects of *Euglena* and paramylon on the gastric mucosal thickness

**Figure 1:**
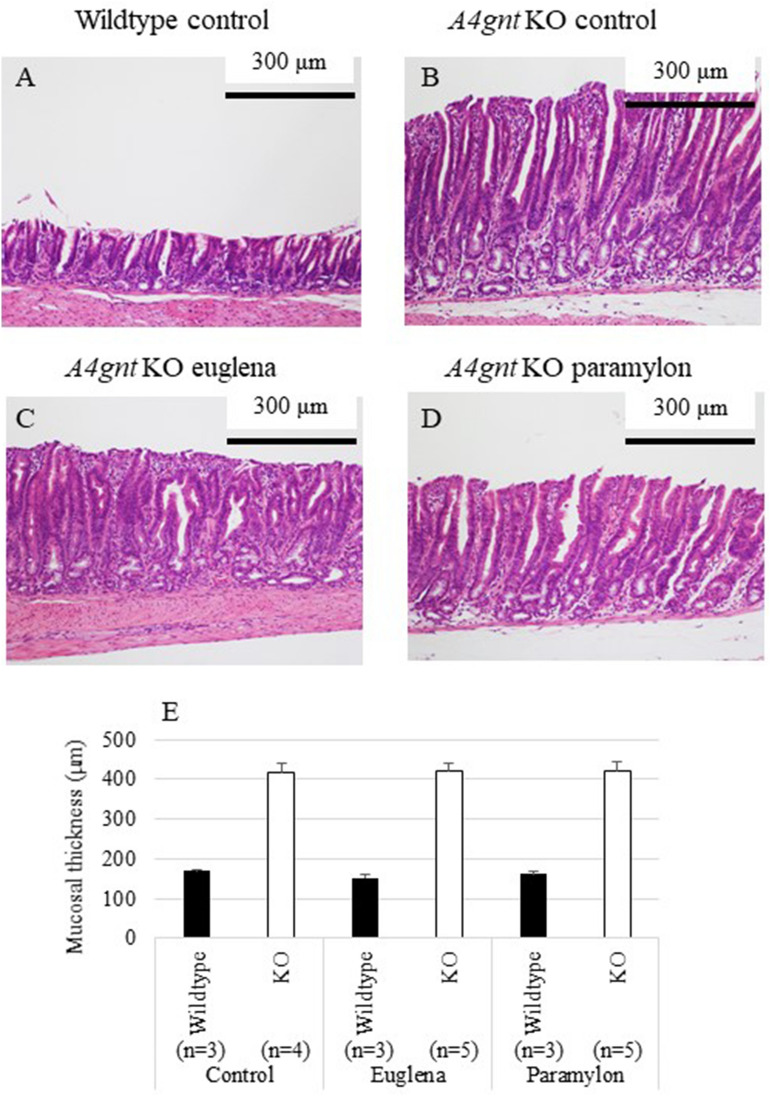
Histopathology of pyloric mucosa of *A4gnt* KO and wildtype mice. (**A**) Wildtype mouse administered saline. (**B**) *A4gnt* KO mouse administered saline. (**C**) *A4gnt* KO mouse administered 50 mg/day of *Euglena*. (**D**) *A4gnt* KO mouse administered 50 mg/day of paramylon. (**E**) Mean ± SD of thickness of pyloric mucosa. The mean thickness of gastric mucosa of 13-week-old *A4gnt* KO mice was significantly thicker compared with that of wildtype mice. Administration of *Euglena* and paramylon did not affect the thickness of the pyloric mucosa.

The body weight change and gross conditions of the mice in all groups were comparable. *A4gnt* KO mice showed significantly thick gastric mucosa in the antrum of the stomach compared with wildtype mice (Fig. 1, Table 1). Administration of *Euglena* and paramylon did not visibly affect the thickness of the pyloric mucosa. The thickness of gastric mucosa of KO control, KO euglena and KO paramylon groups were 418.6 ± 21.4, 421.5 ± 17.5 and 421.8 ± 22.7 µm, respectively (Fig. 1E).

**Table 1:** Summary of statistical analysis.

Groups	Thickness	PMNL	CD3	BrdU	CD31	*Il11*	*Il10*	*Hgf*	*Il1b*	*Cxcl1*	*Ccl2*	*Egf*	*Fgf7*	*Ptg2*
***A4gnt* KO vs. wild type**														
Control	***	*	***	**	***	***	ns	***	***	***	***	***	**	*
Euglena	***	ns	***	*	*	ns	ns	***	ns	ns	*	***	**	*
Paramylon	***	*	*	**	*	*	ns	***	*	*	*	***	*	**
**Control vs. euglena**														
*A4gnt* KO	ns	ns	**	ns	ns	**	ns	ns	ns	*	ns	ns	ns	ns
Wild type	ns	ns	ns	ns	ns	ns	ns	ns	ns	ns	ns	ns	ns	ns
**Control vs. paramylon**														
*A4gnt* KO	ns	ns	**	ns	ns	*	ns	ns	ns	ns	*	ns	ns	ns
Wild type	ns	ns	ns	ns	ns	ns	ns	ns	ns	ns	ns	ns	ns	ns
**Euglena vs. paramylon**														
*A4gnt* KO	ns	ns	ns	ns	ns	ns	ns	ns	ns	ns	ns	ns	ns	ns
Wild type	ns	ns	ns	ns	ns	ns	ns	ns	ns	ns	ns	ns	ns	ns

Tukey’s honest significance test using R software ver. 3.6.2 ^28^ was used to analyze the differences and measure the significance. ***P value < 0.001, **P value < 0.01, *P value < 0.05, ns *P* value > 0.05.

### Effects of *Euglena* and paramylon on the infiltration of inflammatory cells

**Figure 2:**
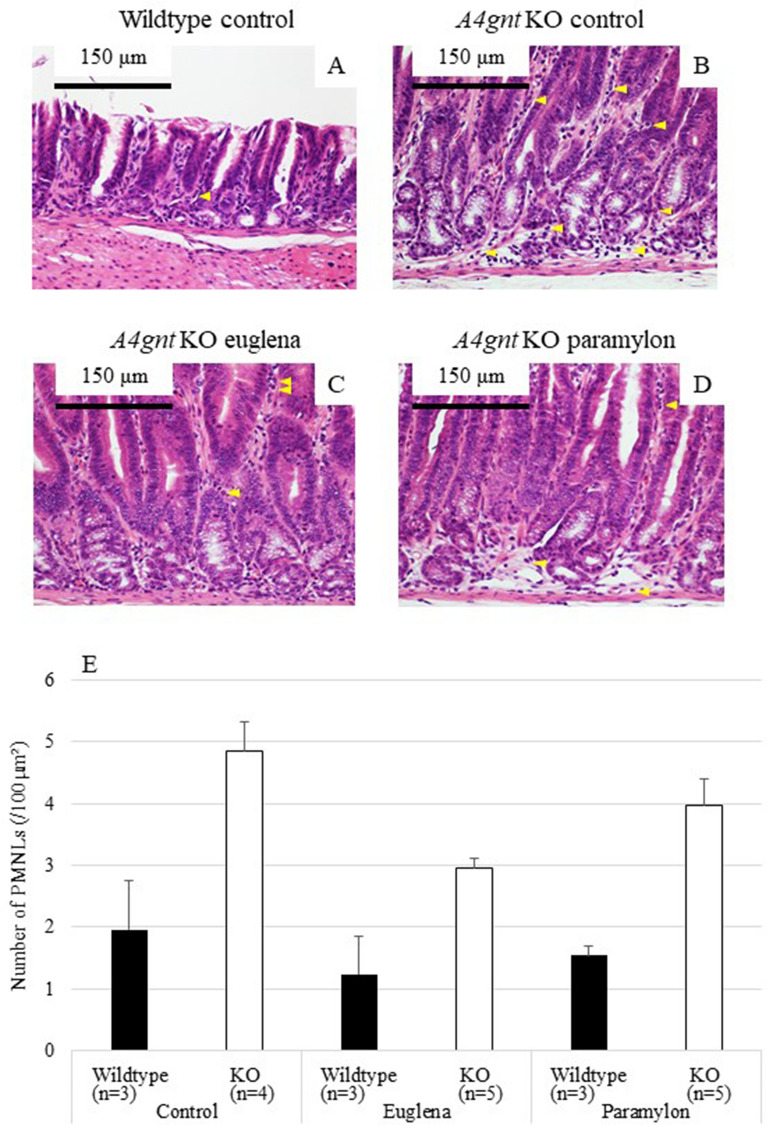
Numbers of PMNLs in pyloric mucosa of *A4gnt* KO and wildtype mice. (**A**) Wildtype mouse administered saline. (**B**) *A4gnt* KO mouse administered saline. (**C**) *A4gnt* KO mouse administered 50 mg/day of *Euglena*. (**D**) *A4gnt* KO mouse administered 50 mg/day of paramylon. Arrowheads indicate PMNLs. (**E**) Mean ± SD of numbers of PMNLs in pyloric mucosa. The mean number of PMNLs in gastric mucosa of *A4gnt* KO mice was significantly more than that of wildtype mice. Administration of *Euglena* significantly reduced the number of PMNLs.

**Figure 3:**
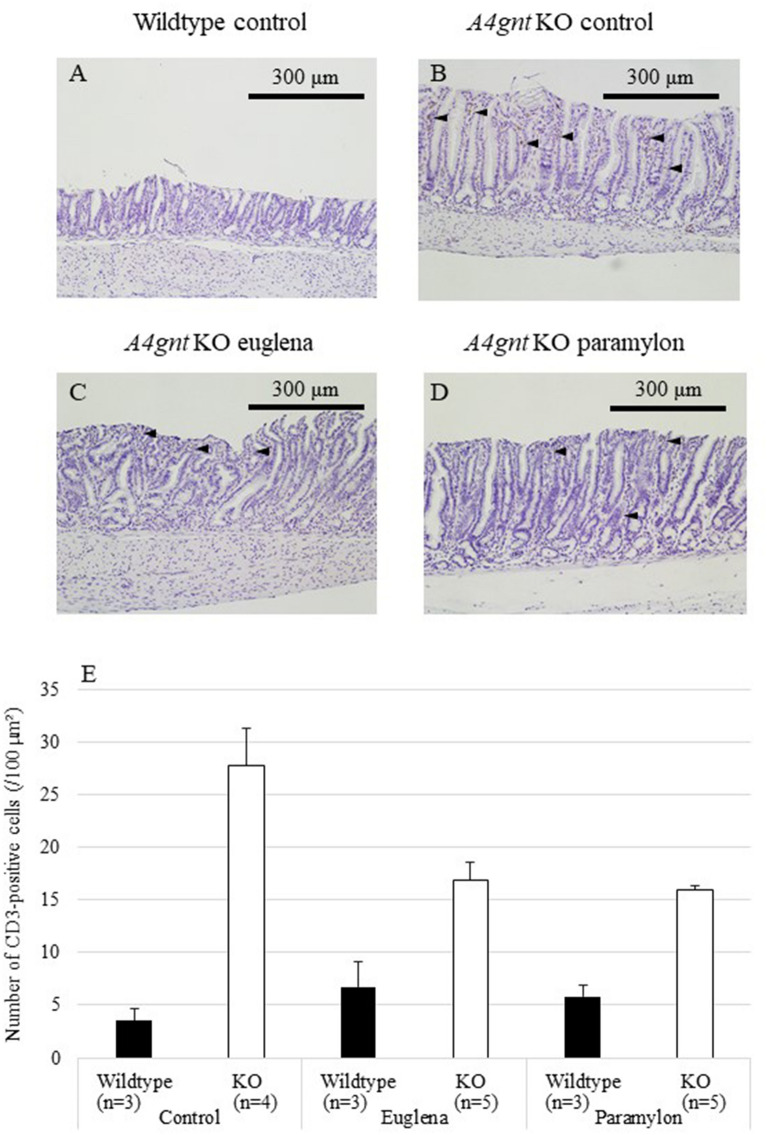
Numbers of CD3-positive lymphocytes in gastric mucosa of *A4gnt* KO and wildtype mice. (**A**) Wildtype mouse administered saline. (**B**) *A4gnt* KO mouse administered saline. (**C**) *A4gnt* KO mouse administered 50 mg/day of *Euglena*. (**D**) *A4gnt* KO mouse administered 50 mg/day of paramylon. CD3-positive cells were stained brown by diaminobenzidine tetrahydrochloride. (**E**) Mean ± SD of numbers of CD3-positive cells in pyloric mucosa. Arrowheads indicate CD3-positive cells. The mean number of CD3-positive cells in gastric mucosa of *A4gnt* KO mice was significantly more than that of wildtype mice. Administration of *Euglena* and paramylon significantly reduced the number of CD3-positive cells of *A4gnt* KO mice compared with those in control group administered with saline.

In *A4gnt* KO mice, it has been strongly suggested that infiltration of inflammatory cells to gastric mucosa initiate the precursor lesion and progress the cascade to differentiated-type gastric cancer^19^. Therefore, we histopathologically assessed the number of infiltrating polymorphonuclear leukocytes (PMNLs) and CD3-positive lymphocytes. The number of PMNLs (Fig. 2A–D, arrowheads) in the pyloric mucosa of *A4gnt* KO mice was significantly higher than that in wildtype mice (Fig. 2E, Table 1), and the number was slightly reduced by *Euglena* administration (*P* = 0.092), thus making the difference between KO euglena group and wildtype euglena group not significant (Table 1). On the other hand, the effect of paramylon on the infiltration of PMNLs was not obvious. The number of infiltrating CD3-positive T-lymphocytes was significantly higher in KO mice compared with in wildtype mice, and significantly reduced by the administration of *Euglena* and paramylon (Fig. 3, Table 1, Supplemental Fig. 1A–C).

### Effects of *Euglena* and paramylon on the proliferation of S-phase cells and angiogenesis

**Figure 4:**
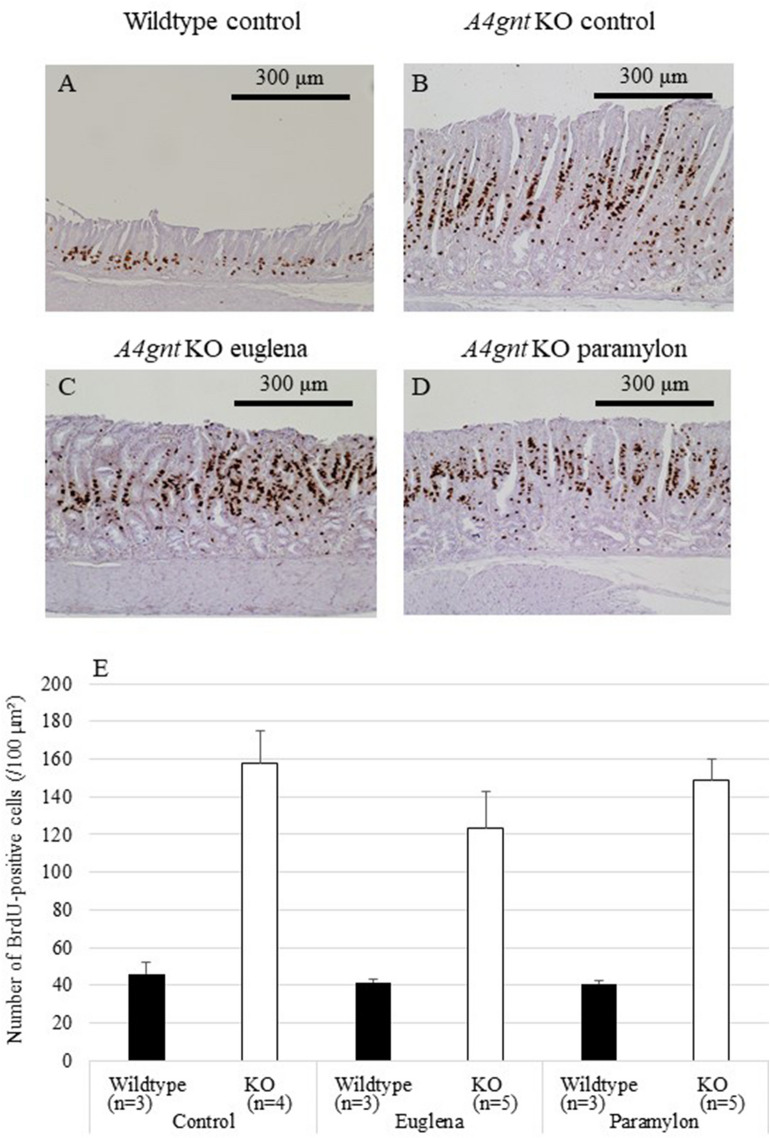
Numbers of BrdU-positive cells in gastric mucosa of *A4gnt* KO and wildtype mice. (**A**) Wildtype mouse administered saline. (**B**) *A4gnt* KO mouse administered saline. (**C**) *A4gnt* KO mouse administered 50 mg/day of *Euglena*. (**D**) *A4gnt* KO mouse administered 50 mg/day of paramylon. BrdU-positive cells were stained brown by diaminobenzidine tetrahydrochloride. (**E**) Mean ± SD of numbers of BrdU-positive cells in pyloric mucosa. The mean numbers of BrdU-positive cells in gastric mucosa of *A4gnt* KO mice was significantly more than that of wildtype mice. Administration of *Euglena* and paramylon did not affect the numbers of these cells.

**Figure 5:**
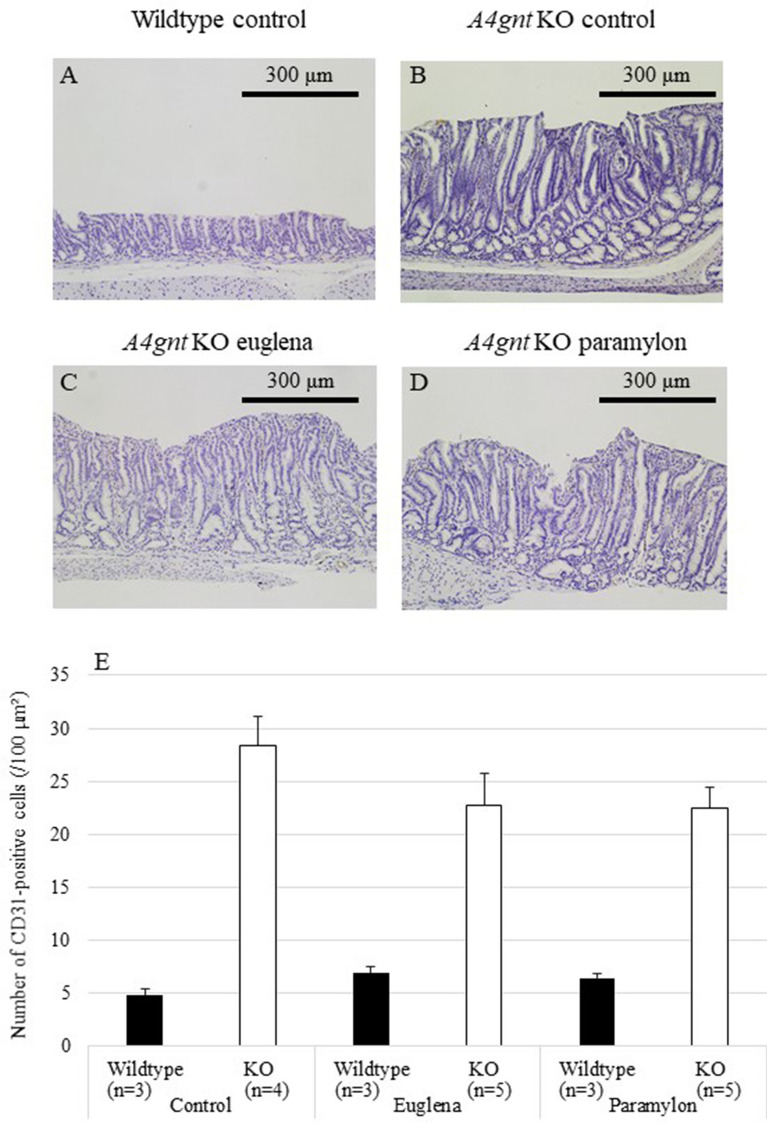
Numbers of CD31-positive cells in gastric mucosa of *A4gnt* KO and wildtype mice. (**A**) Wildtype mouse administered saline. (**B**) *A4gnt* KO mouse administered saline. (**C**) *A4gnt* KO mouse administered 50 mg/day of *Euglena*. (**D**) *A4gnt* KO mouse administered 50 mg/day of paramylon. CD31-positive cells were stained brown by diaminobenzidine tetrahydrochloride. (**E**) Mean ± SD of numbers of CD31-positive cells in pyloric mucosa. The mean numbers of CD31-positive cells in gastric mucosa of *A4gnt* KO mice was significantly more than that of wildtype mice. Administration of *Euglena* and paramylon did not affect the numbers of these cells.

To investigate the possible effects of *Euglena* and paramylon administration on the cell proliferation and angiogenesis, important mechanisms in the gastric carcinogenesis of *A4gnt* KO mice, we then performed BrdU-pulse labeling and CD31 immunohistochemistry (Figs. 4 and 5). The numbers of BrdU- and CD31-positive cells were significantly higher in *A4gnt* KO mice (Figs. 4E and 5E, Table 1). However, the effects of *Euglena* and paramylon administration were comparable with the corresponding control counterparts (Figs. 4 and 5).

### Effects of *Euglena* and paramylon on the gene expression profiles of several cytokines

**Figure 6:**
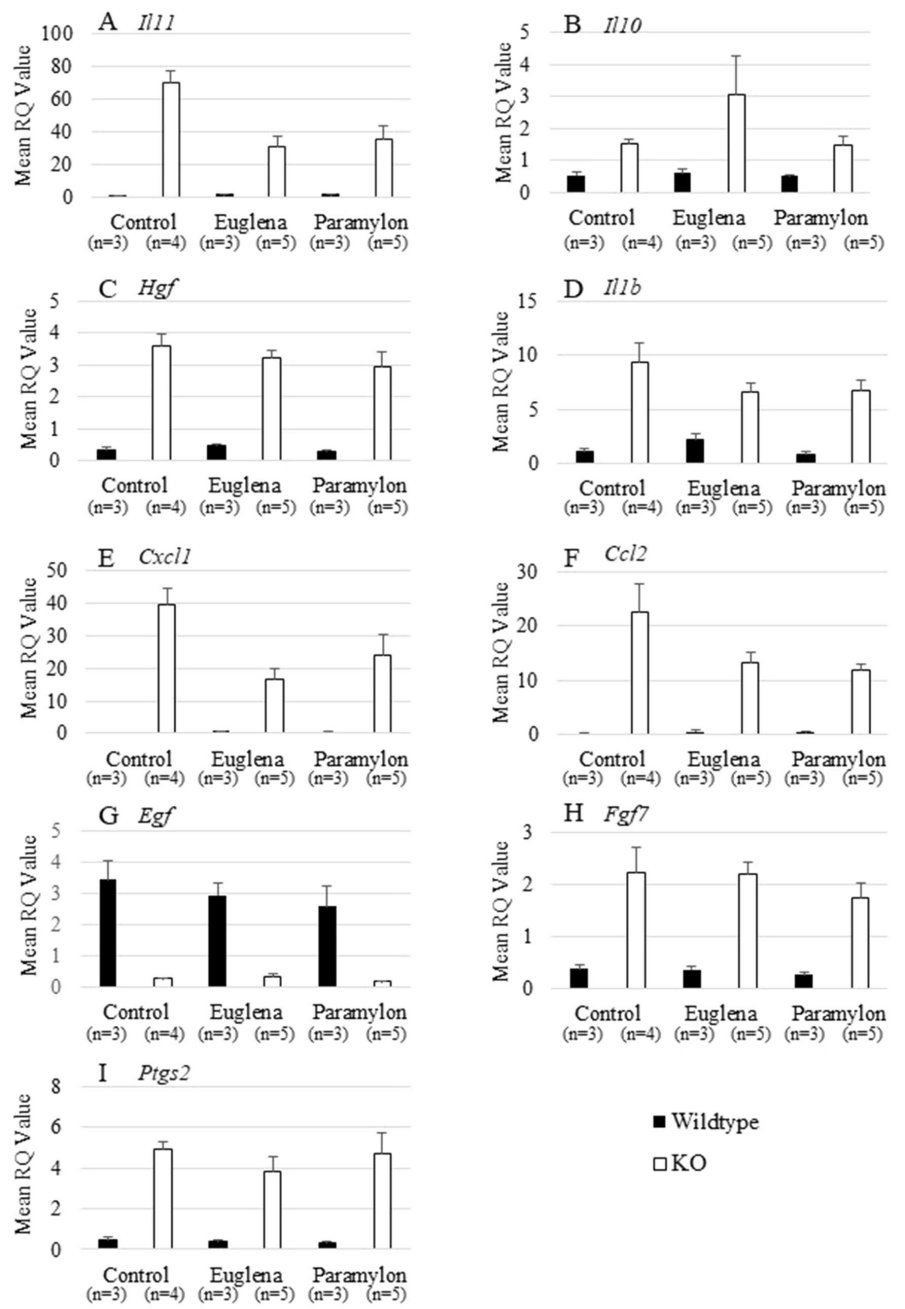
Mean gene expression levels of selected cytokines whose expressions are reported to be substantially altered following *A4gnt* KO-induced gastric carcinogenesis. (**A**) *Il1*, (**B**) *Il10*, (**C**) *Hgf*, (**D**) *Il11b*, (**E**) *Cxcl1*, (**F**) *Ccl2*, (**G**) *Egf*, (**H**) *Fgf7*, (I) *Ptgs2*. The expressions of gene studied were significantly higher in *A4gnt* KO mice than those in wildtype mice except for the expression of *Egf* whose expression was significantly lower in *A4gnt* KO mice. Administration of *Euglena* and paramylon significantly reduced the expressions of *Il11*, *Cxcl1* and *Ccl2*.

Next, we performed quantitative RT-PCR analysis of genes encoding inflammatory chemokine ligands, proinflammatory cytokines, and growth factors, which are either upregulated or downregulated in the *A4gnt* KO at 10 weeks of age^19^. Quantitative RT-PCR analysis revealed marked changes of the mean expression levels of genes examined except for *Il10* in *A4gnt* KO mice compared with those in wildtype mice (Fig. 6, Table 1). In consonance with previous finding^19^, the expressions of *Il1b*, *Il11*, *Cxcl1*, *Ccl2*, *Hgf*, *Fgf7* and *Ptgs2* were significantly higher in *A4gnt* KO mice than in wildtype mice, and *Egf* was significantly lower in *A4gnt* KO mice. Administration of *Euglena* significantly downregulated the expressions of *Il11* and *Cxcl1* in *A4gnt* KO mice when compared with saline-treated *A4gnt* KO mice whereas the gene transcription level of *Cxcl1* between *A4gnt* KO and wildtype mice was insignificant. On the other hand, the gene expression of *Ccl2* of KO euglena group was also appreciably lower than that of KO control group, though the difference did not attain statistical significance. Meanwhile, administration of paramylon to *A4gnt* KO mice also significantly reduced *Il11* and *Ccl2* expressions. In the wildtype control mice, neither *Euglena* nor paramylon solicited any substantial difference in the resulting gene transcription profile of all examined inflammation-associated genes.

### Effects of *Euglena* and paramylon on IgA production in small intestine

**Figure 7:**
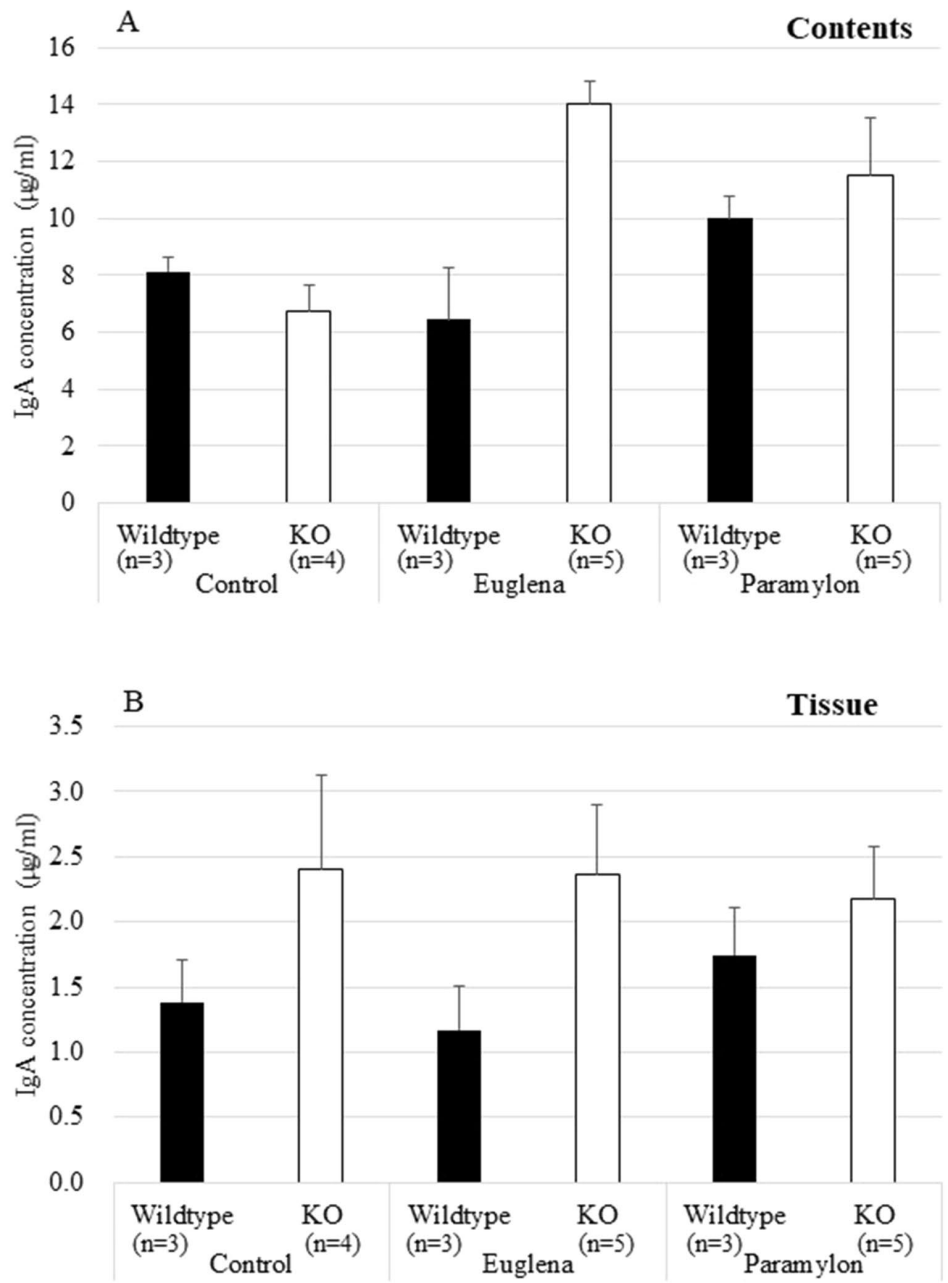
Concentrations of IgA in small intestinal contents and tissue. (**A**) Contents. (**B**) Tissue. In the small intestinal contents, IgA concentration significantly increased after 3 weeks administration of *Euglena* and slightly increased by paramylon administration. In the small intestinal tissue, IgA concentration tend to increase in *A4gnt* KO mice.

The concentration of IgA in the small intestinal contents of *A4gnt* KO mice tended to increase following *Euglena* administration (Fig. 7, Table 1). Nevertheless, we could not find any difference in the concentration of IgA in the small intestinal tissue among the examined treatment groups.

## Discussion

In the present study, we investigated the protective effects of oral administration of *Euglena* and paramylon on gastric dysplasia development using *A4gnt* KO mice, a unique animal model for gastric cancer that spontaneously develops differentiated-type gastric adenocarcinoma in a hyperplasia-dysplasia-carcinoma sequence^19^.

The histopathological and immunohistochemical investigation demonstrated that administration of *Euglena* significantly reduced the elevated infiltration of CD3-positive T-lymphocytes in the pyloric mucosa of *A4gnt* KO mice. Although not statistically significant with the corresponding KO control group, *Euglena* supplementation slightly inhibited the *A4gnt* KO-induced sequestration of PMNLs. In support of this data, we then demonstrated that *Euglena* administration significantly suppressed *Il11* and *Cxcl1* gene expressions, together with a tendency to reduce *Ccl2* expression. IL-11, a member of the IL-6 family, promotes the progression of chronic inflammation to gastric carcinoma via gp130-mediated activation of STAT3 and STAT1 signaling in human gastric carcinogenesis^20^. On the other hand, *Cxcl1-* expressing cancer cells stably exhibited an increased migration and invasion abilities^21^. Owing to the notion that chronic inflammation is the first crucial event in carcinogenesis^22^ that is intricately linked with the continuum of precancerous cascade (metaplasia, dysplasia) to invasive carcinoma^21,23^, our findings altogether suggest that *Euglena* may attenuate the early involvement of gastric carcinogenesis in *A4gnt* KO mice through suppression of mucosal inflammation. Moderate stimulation of IgA production in small intestine also partly supports the influence of *Euglena* on the host immunological condition.

Paramylon, one of the major components of *Euglena*, also significantly suppressed the infiltration of CD3-positive T-lymphocytes and downregulated the expression of *Il11* gene in pyloric mucosa of *A4gnt* KO mice. Although the reduction of the gene expression of *Cxcl1* by paramylon, unlike *Euglena*, was not statistically significant, paramylon significantly reduced *Ccl2* expression. These results indicate the anti-inflammatory effects of paramylon.

Paramylon is a member of β-glucans and various effects of β-glucans on the host have been reported^14,15^. For example, marked suppressive effects of laminaran, a β-glucan derived from brown algae, *Eisenia bicyclis*, on the development of gastric dysplasia is reported using the same *A4gnt* KO mouse model^24^. However, the effects of *Euglena* and paramylon were different from those of laminaran. For example, laminaran suppressed the expression of *Il10*, while *Euglena* and paramylon did not. Paramylon is β-1,3-glucan, consists of only glucose (mean, 700 glucose molecules) and a high molecular weight (~ 500 kDa) insoluble β-glucan^8^, while laminaran is low molecular weight (~ 5 kDa) and soluble. The differences in their structure, such as size of molecule, solubility, types and frequency of side chains, might affect the types and strength of physiological functions. Although it is reported that the effects of laminaran is mediated by dectin-1, the present study did not give enough information to elucidate the mechanism of action of *Euglena* and paramylon. Future studies to elucidate the mode of action, e.g. experiments with inhibitor of receptors, are needed.

In the present study, we analyzed the parameters which have been reported to be affected by *A4gnt* deficiency^19^, because the aim of this study is to investigate the effects of *Euglena* and paramylon on the early involvements of the *A4gnt* KO gastric cancer model. However, it is also possible that *Euglena* and paramylon showed, indirectly, anti-inflammatory effects through the other factors. Further experiments studying the expression of other cytokines would be interesting.

Contrary to expectation that the major mechanism of the effects of *Euglena* is due to paramylon, the effects of *Euglena* were at the same level, or even stronger, when compared with the effects of paramylon. As we administered the same dose of *Euglena* and paramylon to *A4gnt* KO mice, and paramylon consists 30–40% of dry weight of *Euglena*, other bioactive components of *Euglena*, such as vitamin C, vitamin E, and β-carotene, could be important for the anti-carcinogenic effects, which can be harnessed for commercial use^25^.

Cancer survival rate is still closely related to the stage of cancer detection. Therefore, it is important, along with early detection, to control the onset and progression of the cancer by taking functional foods routinely. In the present study, we demonstrated the anti-inflammatory effects of *Euglena* and its component, paramylon in *A4gnt* KO mice. As the chronic inflammation is one of the early involvements of this spontaneous gastric cancer model, it is plausible that *Euglena* and paramylon may restrain the progression of early stage of hyperplasia-dysplasia-adenocarcinoma sequence of the *A4gnt* KO mice through ameliorating the ensuing inflammation in the gastric mucosa. However, as *A4gnt* KO mice develop only precancerous lesion, gastric dysplasia, at the age we used in the present study, and we did not investigate the effects of *Euglena* and paramylon on gastric cancer in this study, it is impossible to discuss about cancer-preventing effects of these compounds. Further experiments with longer period for at least 50 weeks until the formation of gastric cancer are needed to prove the anti-carcinogenic effects of these compounds. We are conducting the long-term experiment with this model to investigate the effects of these compounds on cancer formation. In addition, the administration of *Euglena* and paramylon did not appear to have remarkable effects on the thickness of pyloric mucosa and the number of BrdU- and CD31-positive cells. Three-week administration may not be long enough to reduce cell proliferation and angiogenesis. Administration from earlier stage might be warranted to prove the effects of these compounds. Application of other cancer models showing gastric precancerous lesions^26^ should also be discussed.

## Methods

### Animals

Male and female 10-week-old C57BL/6J-background *A4gnt* KO mice^19^, which exhibited a low-grade gastric dysplasia, were bred in the Laboratory of Biomedical Science, Graduate School of Agricultural and Life Sciences, The University of Tokyo. Their age-matched syngenic wildtype C57BL/6J mice were purchased as control animals from CLEA Japan, Inc., (Japan). Animals were housed in standard polycarbonate cages and maintained under 12 h light/dark cycle (8:00 a.m.: 8:00 p.m.). Rodent chow (CMF; Oriental Yeast Co., Ltd, Japan) and water were given ad libitum. All experimental procedures were performed in accordance with the guidelines and approval of the Institutional Animal Care and Use Committee, Graduate School of Agriculture and Life Sciences, The University of Tokyo (Approval No. P18-053). The experiments were performed also in compliance with the ARRIVE guidelines 2.0.

### Preparation of *Euglena* and paramylon

Spray-dried powder of *Euglena* and paramylon were obtained from euglena Co., Ltd. (Japan). The nutritional analysis results of *Euglena* powder were carbohydrates 45.5%, protein 32.3%, and lipid 14.7%. Approximately 70–80% of the carbohydrate content was paramylon. Paramylon is a β-1,3-d-glucan isolated from *Euglena*, and its content varies with culture method^27^. Paramylon powder was prepared as previously described^6^. In brief, cultured *E. gracilis* cells were collected by continuous centrifugation and washed with water. After suspending in water, the cells were broken down by sonication and the insoluble fraction containing paramylon was collected. To remove the lipid and protein, the crude paramylon preparation was treated with 1% sodium dodecyl sulfate (SDS) solution at 95 °C for 1 h, and then at 50 °C for 30 min with 0.1% SDS. Paramylon was collected by centrifugation, and further refined by repeated washing with water, acetone, and ether, sequentially. The obtained paramylon consists of 95.9% carbohydrates, 3.3% moisture, 0.5% lipids, and 0.4% ash, and the survival rates of protein are less than 0.1%.

### Treatment groups

*A4gnt* KO and wildtype animals were randomly assigned into 3 groups, i.e. control group, euglena group and paramylon group. Control groups were administered 0.4 ml of saline. Euglena groups were administered 50 mg of euglena suspended in 0.4 ml of saline. Paramylon groups were administered 50 mg of paramylon suspended in 0.4 ml of saline. All treatments were given daily via oral gavage for 21 consecutive days. The number of animals were 3, 4, 3, 5, 3 and 5 in wildtype control, KO control, wildtype euglena, KO euglena, wildtype paramylon and KO paramylon groups, respectively.

### Histopathology of pyloric mucosa

One hour prior to sacrifice, animals were injected intraperitoneally with bromodeoxyuridine (5-bromo-2-deoxyuridine, BrdU) solution (10 mg/kg) to label proliferating cells in the S-phase of the cell cycle^19^. Animals were euthanatized by cervical dislocation and stomach tissues along with a small portion of the duodenum were harvested. Stomach was cut longitudinally along the greater curvature, washed with 1× PBS and divided into half for subsequent histopathological, immunohistochemical and gene expression analyses. A half of the stomach was fixed in 10% buffered formaldehyde for at least 48 h and subjected to routine paraffin technique. Four-micrometer sections were then prepared and stained with standard H&E. Pyloric mucosal thickness was measured from the base of the gastric mucosal layer up to the highest tip of a properly oriented epithelium at three different areas of the pyloric region. Mean of triplicate measurements for each animal was obtained. For determining the number of infiltrating granulocytes, a defined 100 μm^2^ area with the highest cell density along the pyloric region was counted and a mean of triplicate measurements was then obtained.

### CD3, CD31 and BrdU immunohistochemistry

Formalin-fixed and paraffin-embedded (FFPE) tissue sections (4 μm) were deparaffinized by xylene for 4 times, rehydrated with increasing grades of alcohol (100, 95, 90, 80 and 70%), and washed with Tris-buffered saline (TBS; 0.1 M, pH 7.4) for 3 times. Prior to incubation with primary antibodies, antigen retrieval was carried out using 4 N HCl followed by digestion with 0.5% trypsin (Thermo Fisher Scientific Inc., USA) for 30 min at 37 °C for anti-BrdU whereas incubation in 0.1 M sodium citrate buffer, pH6 for 10 min was done for anti-IL-10, anti-CD3 and anti-CD31. After this, endogenous peroxidase was blocked by 10% H_2_O_2_-methanol solution for 15 min. Blocking of non-specific background staining were accomplished using 8% skimmed milk for CD3 and CD31, and Histofine Mousestain Kit (Nichirei Biosciences, Japan) for BrdU. Tissue sections were then subsequently incubated overnight in a humidified chamber at 4 °C using the following primary antibodies: anti-CD3 (FLEX Polyclonal Rabbit Anti-Human CD3 Ready-to-Use; DakoCytomation, Denmark), anti-CD31 (CD31 Rabbit Polyclonal Antibody Ready-to-Use for Immunohistochemical Staining; Neomarkers, USA) and anti-BrdU (Monoclonal Mouse Anti-Bromodeoxyuridine Clone Bu20a 1:50; DakoCytomation, Denmark). After washing by TBS for 3 times, immunolabeling of tissue sections was performed using Dako EnVision + System-HRP Labelled Polymer Anti-Rabbit (DakoCytomation) for 1 h under room temperature (CD3 and CD31) or universal immuno-peroxidase polymer (Simple Stain Mouse Max PO; Nichirei Biosciences) for 10 min under room temperature after further blocking by Histofine Mousestain Kit (BrdU). Immunoreaction was finally visualized using diaminobenzidine tetrahydrochloride-H_2_O_2_ solution. The number of CD3-, CD31- and BrdU-positive cells was assessed using a defined 100 μm area in the pyloric region with the highest cell density and the mean of 8 measurements was obtained.

### Quantitative RT-PCR

Another half of harvested gastric pyloric tissues were placed in RNAlater (Invitrogen, USA) and stored under − 80 °C until further analysis, and quantitative RT-PCR was performed basically the same as previously described^24^. The samples were homogenized with Shake Master Auto BMS-A20TPver.2.0 (BMS, Japan) and RNA was extracted using Nucleospin RNA isolation kit (Macherey–Nagel, Germany) according to the manufacturer’s instructions. First strand cDNA was synthesized by PrimeScript RT reagent kit (Perfect Real Time; Takara Bio, Japan). Real-Time PCR analysis was accomplished using TB Green *Premix Ex Taq* II kit (TIi RNaseH Plus; TaKaRa Bio, Japan) and StepOnePlus Real-Time PCR (Applied Biosystems, USA) to determine the expression level of the following genes which are reported to be affected by *A4gnt* deficiency in the previous study^19^: *Il1b*, *Il10*, *Il11*, *Cxcl1*, *Ccl2*, *Hgf*, *Egf*, *Fgf7*, *Ptgs2*, and *Actb*. The primers utilized in the present study are shown in Table 2. Reaction was heated at 98 °C for 1 min followed by 40 thermal cycles at 95 °C for 30 s, 95 °C for 5 s, 60 °C for 34 s, and one cycle of melting curve at 95 °C for 15 s, 60 °C for 1 m, and 95 °C for 15 s. Expression of mRNA was normalized to the expression level of the housekeeping gene, *Actb* and determination of comparative CT value was done by setting the average mRNA expression level of C57BL/6J mice as 1.0. Analysis was run in duplicates.

**Table 2:** List of real-time PCR primers.

Genes	Forward	Reverse
*Actb*	AAGTGTGACGTTGACATCCG	GATCCACATCTGCTGGAAGG
*Il1b*	GCAACTGTTCCTGAACTCAACT	ATCTTTTGGGGTCCGTCAACT
*Il10*	GCTCTTACTGACTGGCATGAG	CGCAGCTCTAGGAGCATGTG
*Il11*	TGTTCTCCTAACCCGATCCCT	CAGGAAGCTGCAAAGATCCCA
*Cxcl1*	CTGGGATTCACCTCAAGAACATC	CAGGGTCAAGGCAAGCCTC
*Ccl2*	TTAAAAACCTGGATCGGAACCAA	GCATTAGCTTCAGATTTACGGGT
*Hgf*	ATGTGGGGGACCAAACTTCTG	GGATGGCGACATGAAGCAG
*Egf*	AGCATCTCTCGGATTGACCCA	CCTGTCCCGTTAAGGAAAACTCT
*Fgf7*	CTCTACAGGTCATGCTTCCACC	ACAGAACAGTCTTCTCACCCT
*Ptgs2*	TTCAACACACTCTATCACTGGC	AGAAGCGTTTGCGGTACTCAT

### IgA concentration of small intestinal contents and tissue

Whole small intestine was harvested and the intestinal contents were washed out using a total of 6 ml of cOmplete (Roche, Switzerland) solution dissolved in 50 ml of PBS and then collected. The intestinal contents were centrifuged at 600×*G* for 5 min under 4 °C and the supernatant was centrifuged again at 9200×*G* for 5 min. Two centimeters of ileum about a quarter from the cecum was also collected as small intestinal tissue. The tissue was cut open, washed with PBS and homogenized with 2 ml of homogenization buffer (0.475 g HEPES, 500 µl 1:100 Triton X-100 (Sigma, USA), 5 ml glycerol and 1 tablet of cOmplete in 50 ml). The homogenate was centrifuged at 4000 rpm for 10 min under 4 ℃ and the supernatant was centrifuged again at 7000 rpm for 5 min. IgA concentration of the supernatants were measured with IgA Mouse Uncoated ELISA Kit with Plates (Invitrogen, USA) according to the manufacturer’s instructions at 450 nm using iMark microplate reader (Bio-Rad Laboratories, USA).

### Statistical analysis

All values were expressed as mean ± SD and compared with Tukey’s honest significance test using R software ver. 3.6.2^28^. Values with *P* < 0.05 were considered statistically significant.

## Supplementary Information

Supplementary Information 1.

Supplementary Information 2.

Supplementary Information 3.

Supplementary Information 4.
