# Supplementary material for: Effects of orally administered Euglena gracilis and its reserve polysaccharide, paramylon, on gastric dysplasia in A4gnt knockout mice
Source: Sci Rep. 2021 Jul 1;11:13640. doi: 10.1038/s41598-021-92013-5 (PMC8249615; doi:10.1038/s41598-021-92013-5)
Supplement: Supplementary file 1 — Supplementary Information 1. [file 41598_2021_92013_MOESM1_ESM.docx]

**Supplementary material**

**Effects of orally administered *Euglena gracilis* and its reserve polysaccharide, paramylon, on gastric dysplasia in *A4gnt* knockout mice**

Masataka Iida^1^, Mark Joseph Desamero^2, 3^, Kosuke Yasuda^4^, Ayaka Nakashima^4^, Kengo Suzuki ^4^, James Ken Chambers^5^, Kazuyuki Uchida^5^, Ryohei Ogawa^6^, Satoshi Hachimura^6^, Jun Nakayama^7^, Shigeru Kyuwa^2^, Kozue Miura^1^, Shigeru Kakuta^2*^, Kazuhiro Hirayama^1*^

Legend of supplemental figure

Supplemental Figure 1. Immunohistochemistry of CD3-positive lymphocytes in gastric mucosa of individual wildtype (WT) and *A4gnt* KO (KO) mice. (A) Control mice administered saline. (B) Mice administered 50 mg/day of *Euglena*. (C) Mice administered 50 mg/day of paramylon. CD3-positive cells were stained brown by diaminobenzidine tetrahydrochloride. The framed images are the individuals also shown in Figure 3.
