# Supplementary figures and images for: Effects of orally administered Euglena gracilis and its reserve polysaccharide, paramylon, on gastric dysplasia in A4gnt knockout mice
Source: Sci Rep. 2021 Jul 1;11:13640. doi: 10.1038/s41598-021-92013-5 (PMC8249615; doi:10.1038/s41598-021-92013-5)

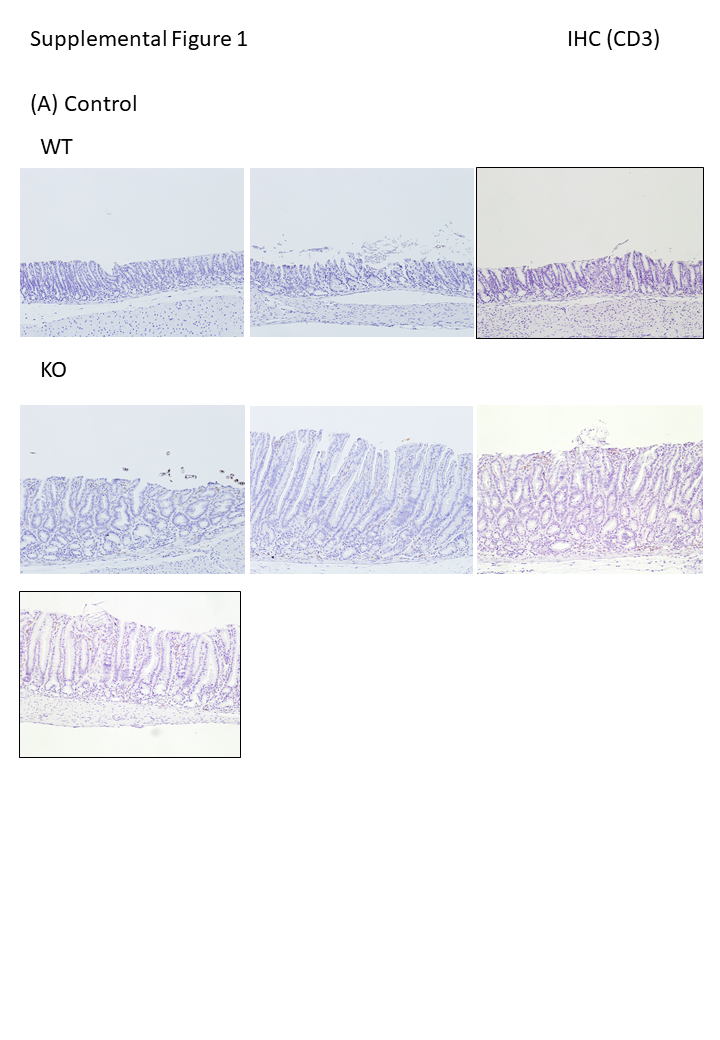

Supplement: Supplementary file 2 — Supplementary Information 2. [file 41598_2021_92013_MOESM2_ESM.tif]

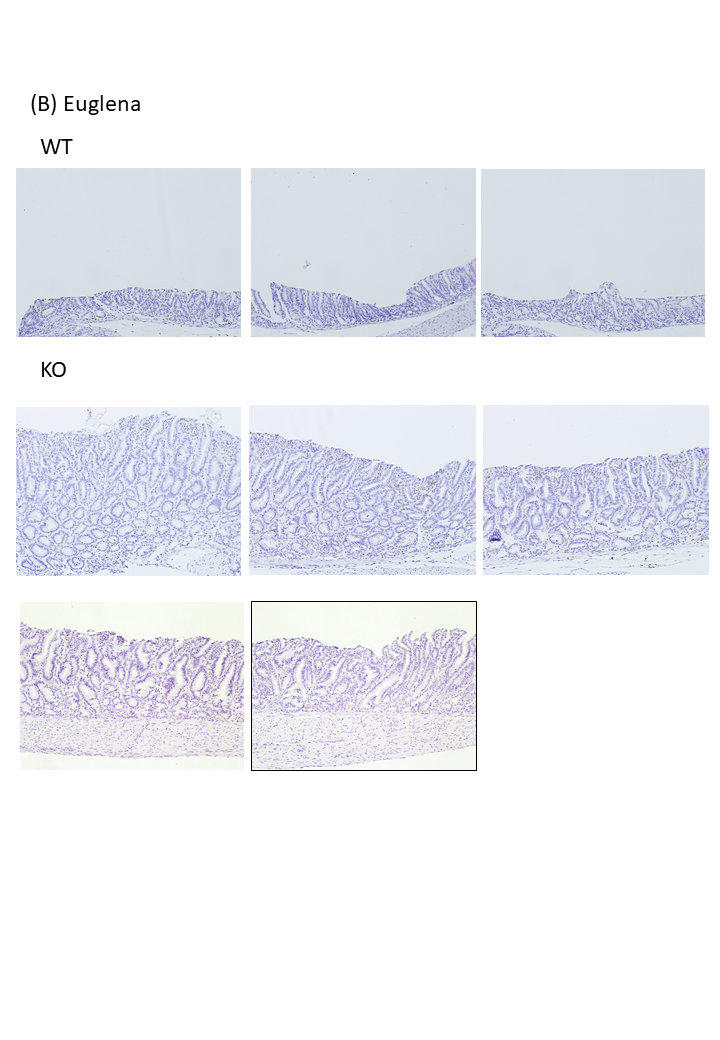

Supplement: Supplementary file 3 — Supplementary Information 3. [file 41598_2021_92013_MOESM3_ESM.tif]

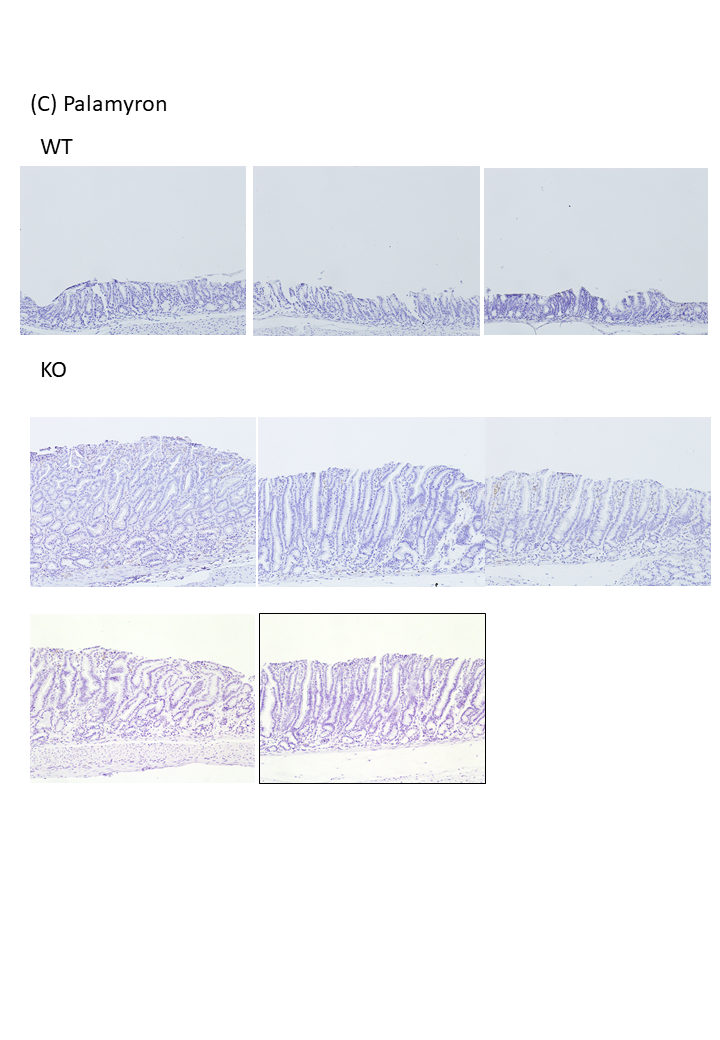

Supplement: Supplementary file 4 — Supplementary Information 4. [file 41598_2021_92013_MOESM4_ESM.tif]
